# Evaluation of low level laser therapy and lidocaine versus chlorohexidine for the management of traumatic oral ulcers in children: a randomized controlled study

**DOI:** 10.1038/s41598-025-34529-8

**Published:** 2026-01-27

**Authors:** Maryam El Mansy, Mohamed Farouk Rashed

**Affiliations:** Researcher of Pediatric Dentistry, Orthodontics and Pediatric Dentistry Department, Oral and Dental Research Institute, National Research Centre, 33 El Buhouth St, Dokki, Giza, 12622 Egypt

**Keywords:** Oral ulcer, Children, Low level laser therapy, Cholorhexidine, Lidocaine, Diseases, Health care, Medical research

## Abstract

This study aimed to compare the effectiveness of Low-Level Laser Therapy (LLLT), lidocaine (LC) gel, and chlorhexidine (CHX) mouthwash in the management of traumatic oral ulcers in children, with respect to ulcer size, pain relief and parental satisfaction. Thirty children aged 6–14 years with clinically diagnosed traumatic oral ulcers were randomly allocated into three groups (*n* = 10 each): Group I (LLLT), Group II (LC), and Group III (CHX). Ulcer size was measured using a calibrated periodontal probe, pain intensity was assessed with the Visual Analogue Scale (VAS) and parental satisfaction was evaluated using a Likert scale. Ulcer size reduction was greatest in Group I, followed by Group II and Group III, with statistically significant differences observed at day 5 (*p* = 0.0001) and day 7 (*p* = 0.0072). Group I also recorded the lowest VAS scores immediately, and at 2, 5, and 7 days (*p* = 0.0001) compared with the other groups. Parental satisfaction was significantly higher in the LLLT group compared with the LC and CHX groups (*p* = 0.006). LLLT demonstrated superior effectiveness in accelerating healing by reducing related size and pain in traumatic oral ulcers compared with LC gel and CHX mouthwash. Despite CHX being the standard approach for oral ulcer management, it showed the lowest efficacy. Parental acceptance of LLLT was high, with minimal discomfort related to treatment visits, supporting its potential as a favourable treatment modality in paediatric dental practice.

**Trial registration: **On August 16, 2025, this study was retrospectively registered on ClinicalTrials.gov under the identification number NCT07138586 with URL; https://clinicaltrials.gov/study/NCT07138586.

## Introduction

 Oral ulcers are defined as a complete discontinuity of the oral epithelial lining, typically covered by a fibrinous membrane and presenting clinically as a whitish lesion with an erythematous margin^1^. Clinically, these lesions may be categorized as acute, chronic, or recurrent. Ulcers that persist for two weeks or longer are generally considered as chronic or potentially malignant, whereas those that resolve within a shorter duration are classified as acute ones. Recurrent ulcers, by contrast, are characterized by repeated episodes and separated by symptom-free intervals with spontaneous healing in between^2,3^.

Traumatic injury to the oral mucosa is among the most frequent causes of ulceration. Chen and colleagues (2010) reported that the buccal mucosa was the most commonly affected site (42%), followed by the tongue (25%) and the lower lip (9%)^4^. Traumatic ulcers arise from physical damage to the oral mucosa, commonly resulting from sharp food particles, accidental lip or cheek biting during mastication, or burns of thermal, electrical, or chemical origin. Post-dental treatment trauma has also been reported as a particularly frequent cause of this problem^5^.

Previous investigations have shown that approximately 13% of children aged between 2 and 18 years sustain soft-tissue injury following unilateral or bilateral mandibular nerve block anaesthesia. The highest prevalence of this incident has been observed in younger children, with incidences of 18% in those under 4 years of age, 16% in children aged 4–7 years, 13% in those aged 8–11 years, and 7% among those older than 12 years. Additional causes of iatrogenic traumatic ulcers include accidental trauma from sharp dental instruments or inadvertent contact with the high-speed handpiece^6^.

Clinically, traumatic ulcers are typically presented as lesions with a yellowish-white necrotic pseudomembrane surrounded by erythematous, slightly elevated margins. Pain usually decreases within the first week, and most lesions resolve spontaneously within 14 days unless healing is delayed by secondary infection or continued mechanical or dietary irritation^7^.

Topical management of oral ulcers aims to prevent secondary infection, relieve pain, reduce inflammation, and promote healing. Chlorhexidine (CHX) mouthrinse has demonstrated potential activity against several enveloped viruses, including Herpes Simplex Virus, Cytomegalovirus, Influenza, and Respiratory Syncytial Virus. In addition, topical antibiotics such as doxycycline and minocycline have been reported as effective options for these ulcers. Bioadhesive pastes containing 20% benzocaine provide symptomatic pain relief by forming a protective coating, whereas lidocaine (LC) gel offers short-term analgesia. Furthermore, topical diclofenac (3% combined with hyaluronic acid 2.5%) has been shown to exert potent anti-inflammatory effects^3,8^.

Lidocaine is a widely used, safe, and effective agent for local anaesthesia. Since its initial patent in 1948, its pharmacological properties have been extensively investigated, with much of the evidence consolidated during the 1970s. Currently, LC is used for topical anaesthesia of the skin and oral mucosa as well as for infiltration and nerve block techniques. It is also available over the counter for symptomatic management of oral ulcers; however, robust evidence from clinical trials in this context remains limited^9^.

In recent years, low-level laser therapy (LLLT) has gained increasing attention for its enhanced wound-healing, analgesic, and anti-inflammatory potential, relevant to the management of oral ulcers and other pathological conditions. LLLT involves the emission of light at a single wavelength within the visible to near-infrared spectrum, producing non-thermal, photobiomodulatory effects at the cellular level. Once absorbed by target cells, the photons initiate a cascade of biochemical processes that stimulate tissue repair and accelerate wound healing^10^.

The mechanism of LLLT in relieving pain may be attributed to the stimulation of release of serotonin and endorphins which produce an analgesic effect together with the decrease in membrane permeability of the nerve cell membrane for NA and K. Moreover, it reduces prostaglandin synthesis which produces anti-inflammatory effects with improved blood circulation to the oral mucosa^11–13^.

Among LLLT devices, diode lasers are of particular interest due to their compact size, broad wavelength range, and compatibility with fibre-optic delivery. Their application is associated with biomodulatory and analgesic benefits without inducing thermal damage or tissue ablation^14,15^.

To the best of our knowledge, only a limited number of randomised controlled trials have compared the efficacy of LLLT with that of conventional topical therapies in the management of traumatic oral ulcers in children. Therefore, the aim of the present study was to compare the healing of traumatic ulcers in paediatric patients with respect to pain reduction and ulcer size when treated with LLLT (Group 1), LC gel (Group 2), or CHX mouthwash (Group 3, control). In addition, parental satisfaction with the three treatment modalities was assessed and quantified before and after therapy.

The null hypothesis states that there will be no significant difference among LLLT, LC gel, and CHX mouthwash with regard to ulcer size reduction, pain relief, or parental satisfaction.

## Materials and methods

### Study design

**Fig. 1:**
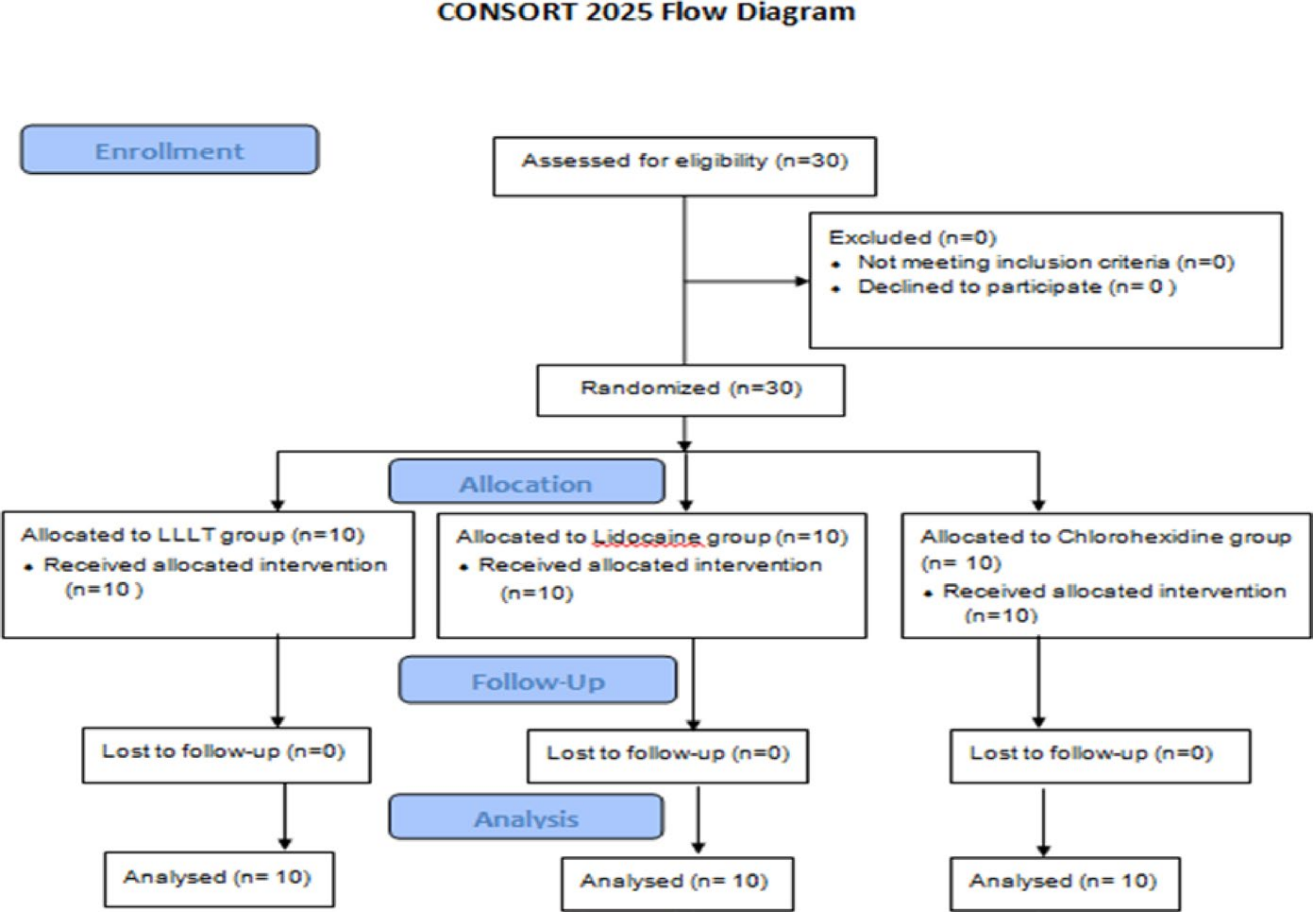
Flowchart shows participants’ enrollment, randomization, allocation, follow-up and analysis in accordance to CONSORT principles.

This study was conducted in accordance with the Consolidated Standards of Reporting Trials (CONSORT) 2025 statement for the design and reporting of clinical trials^16^. Thirty children diagnosed with traumatic oral ulcers were randomly assigned to three groups of ten participants each at baseline. The allocated interventions were subsequently administered, and outcomes were evaluated at predetermined time points. A detailed outline of participant enrolment, allocation, follow-up, and analysis is presented in the CONSORT 2025 flow diagram (Fig. 1).

### Ethical approval and registration

The Medical Research Ethical Committee, National Research Centre, Egypt, granted the approval for this research on September 24, 2024, under the number 0342. The ethical standards of Helsinki, as outlined in the 1964 and subsequent amendments were adhered to strictly^17^.On August 16, 2025, this study was retrospectively registered on ClinicalTrials.gov under the identification number NCT07138586 with URL; https://clinicaltrials.gov/study/NCT07138586.

### Sample size calculation

According to a previous study by Bardellini et al., the mean of Visual Analogue Scale (VAS) after 4 days varied between 1 ± 0.72, and 3 ± 1.38^18^. Using G power statistical power Analysis program (version 3.1.9.4) for sample size determination, a total sample size (*n* = 30; subdivided to 10 in each group) was sufficient to detect a large effect size (f) = 0.61, with an actual power (1-β error) of 0.8 (80%) and a significance level (α error) 0.05 (5%) for two-sided hypothesis test^19^.

### Participants

Thirty children of both sexes, aged between 6 and 14 years and diagnosed with traumatic oral ulcers, were recruited for this study. All participants were referred from the outpatient dental clinic of the National Research Centre and were enrolled according to the following eligibility criteria:

#### Inclusion criteria

Children were eligible to participate if they met all of the following conditions:

1. Aged between 6 and 14 years, with no apparent medical conditions.
2. Participants presenting with traumatic ulcers associated with an identifiable local cause, such as but not limited to; a history of a dental procedure performed on the same day or within the 24 h preceding ulcer onset, Presence of a sharp restoration or presence of orthodontic appliances or other intraoral prosthetic devices etc….
3. The ulcer must have appeared on the same day, or no more than 24 h before the patient presented to the clinic.
4. No prior treatment with any modality for the ulcer.
5. Willingness to participate in the clinical study.
6. Good oral hygiene.

#### Exclusion criteria

Participants were excluded if they met any of the following conditions:

1. Presence of psychological or psychiatric disorders.
2. Current use of anticoagulant, anti-inflammatory, or immunosuppressive medications.
3. History of systemic disease (endocrine–metabolic disorders, rheumatological conditions, hormonal disturbances, or immunodeficiency) or current use of corticosteroid-based therapy.
4. Lack of cooperation from the child or parents.
5. Irregular attendance at follow-up visits, which precluded adequate evaluation^20^.

### Setting and location

Patient recruitment was conducted from November 2024 to January 2025, and the follow-up period for last cases ended in February 2025.

### Intervention

#### Informed consent

Prior to the intervention, parents read thoroughly and subsequently signed the informed permission after the clinicians provided a simplified summary of the procedures. A verbal assent was taken from the child before beginning of any procedure.

#### Randomization

The children participated in the current study were allocated into three groups (*n* = 10) at a 1:1 allocation ratio. Randomization was done on 15 November 2024 via the website www.random.org. Children were allocated into three groups along with the applied treatment modality; Group one: LLLT, Group two: LC gel (Oracure oral gel^®^) and Group three: CHX mouthwash (Orovex^®^; control group).

#### Allocation concealment

External investigator, not involved in the clinical steps of the trial performed the random sequence generation using software available at http://www.random.org. The randomization table was securely maintained by this investigator to ensure complete allocation concealment. M.F. enrolled the participants, and M.E. revealed the group allocation for each child at the time of treatment. M.E. administered the assigned interventions. M.F. was responsible for outcome assessment.

#### Blinding

This study followed a double-blind design, as both the outcome assessor and the biostatisticians were unaware of the intervention allocated to each participant. Complete blinding was not feasible for the operator, parents, and children due to the distinct nature of the intervention procedures.

#### Clinical procedures

1. **Pre-study assessment: **Comprehensive pre-operative data were collected, including the child’s name, age, gender, medical and dental history, as well as the occupation and educational level of their parents.
2. **Diagnosis of traumatic oral ulcer: **Baseline ulcer characteristics were documented. The onset, duration, and severity of pain were assessed using the (VAS). A clear history of trauma preceding the ulcer’s appearance was obtained to confirm diagnosis. Ulcer size was measured at baseline using the standardized ruler method.
3. **Grouping of samples: **

- Group one: children received LLLT using diode laser 980 nm.
- Group two: children were instructed to use LC gel (Oracure gel^®^) 4 times daily and.
- Group three: children were instructed to use CHX mouthwash (Orovex^®^) 4 times daily (control group).

(4) **Treatment modalities: **

- **Group one: **the oral ulcer was treated with the application of (LLLT) using diode laser 980 nm (Doctor Smile, Wiser, LAMBDA SpA, Italy).

Diode Laser criteria: 980 nm wavelength, and 0.3 W output power and 18 J/ Cm^2^ Fluence per point was applied in a continuous wave emission mode for 60 s using a Flattop hand piece model AB 2799 (delivered with a flattop beam profile hand-piece (Doctor Smile, Lambda, Vincenza, Italy) with a Beam spot size at target one Cm^2^^21^.

The tip was perpendicularly directed away from the lesion by 1 mm in a noncontact mode. Children and clinicians wore protective eyeglasses matched to our laser wavelength to avoid radiation hazards.

The sessions were as follows; at baseline, after two days, five days and seven days.

- **Group two: **the children were given a tube of LC oral gel (Oracure oral gel^®^, 30 gm, Amoun Pharmaceutical Industries Company).
- Active ingredients: each 100 gm of the gel contains Lidocaine HCl 2.0 gm and Cetylpyridinium chloride 0.1 gm.

At baseline, the oral gel was applied topically to the affected area by the clinician using a clean, gloved fingertip. Following this initial application, both the child and their parents were instructed and encouraged to continue applying the gel in the same manner four times daily for six consecutive days. Detailed written and verbal instructions were provided during the initial visit to ensure that parents fully understood the correct method and frequency of use for both products.

Children were advised to refrain from eating or drinking for at least one hour following each application and to avoid the consumption of spicy foods for a period of two weeks.

- **Group three: **the children were given a bottle of CHX mouthwash (Orovex^®^ mouthwash, Macro Group Pharmaceuticals, Egypt).
- Active ingredients: (2% CHX, Fluoride and thymol).

At baseline, the child was instructed to gargle with 20 ml (one capful) undiluted mouthwash then swish for 30 to 60 s before spitting it. This procedure should be repeated 4 times per day for 6 days. The child and their parents were motivated to adhere to these instructions. The child was also instructed not to eat or drink for 1 h and avoid spicy food for 2 weeks.

After that the children’s ulcers were evaluated immediately, after two, five, seven days and two weeks. Regarding LC and CHX groups, regular use of both medications was confirmed at each follow-up visit.

(5) Outcome assessment:

A) Subjective pain assessment of the ulcer

**Fig. 2:**
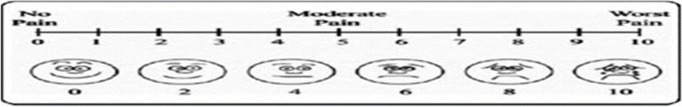
an image shows different scales of VAS.

Pain severity was evaluated using the (VAS), which consisted of a 10-cm horizontal line illustrated with simple images and numerical scores ranging from 0 (no pain) to 10 (severe pain). Children were asked to indicate their pain level by marking the point on the line that best represented their experience^22^. Assessments were performed at baseline (preoperatively), immediately after the intervention, and subsequently at two, five, and seven days, as well as at two weeks. The VAS tool is illustrated in Fig. 2.

B) Objective assessment of the ulcer sizes

The size of the index ulcer was recorded at baseline (preoperatively), immediately after the intervention, and at two, five, and seven days, as well as at two weeks. Ulcer dimensions were measured using the ruler method with a calibrated periodontal probe (University of Michigan 0 probe). For oval-shaped lesions, the maximum and minimum diameters were recorded, and the two measurements were subsequently multiplied to calculate the lesion’s cross-sectional area^3^.

C) Parental acceptance of the therapy

At the end of treatment, parental acceptability was evaluated using a structured questionnaire. Parents were asked to record the level of comfort of their children with the therapy provided and the extent of inconvenience related to attending multiple appointments. Responses were measured on a five-point Likert scale, where 1 represented “unsatisfied”, 2 represented “low acceptance” 3 represented “medium acceptance”, 4 represented “satisfied” and 5 represented “very satisfied” regarding parental acceptance of the procedure Similarly answers for discomfort for the need of appointments were ranked, where 1 represented “unsatisfied”, 2 represented “low acceptance” 3 represented “medium acceptance”, 4 represented “acceptable” and 5 represented “no problems”^18^.

The questionnaire, administered in Arabic, comprised two sections. The first section collected demographic information of the participating parents, while the second one explored their acceptance of the treatment modalities. In Group One, parents rated their satisfaction with laser therapy and the degree of discomfort associated with repeated clinical visits. In Group two and three, parents assessed the acceptability of the taste and frequency of application of the prescribed oral gel or mouthwash. All questionnaires were completed in the clinic waiting area; where parents were given sufficient time to provide considered responses^18^.

**Fig. 3:**
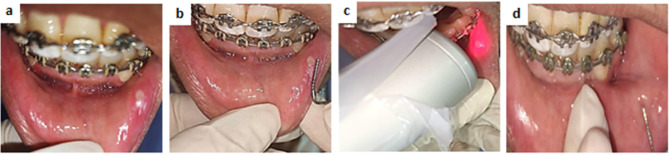
Clinical photos of a case of LLLT group illustrating the healing process at different time points; (**a**) pretreatment (baseline), (**b**) at day2 (**c**) during application of laser using flat top hand piece (**d**) at day 5.

**Fig. 4:**
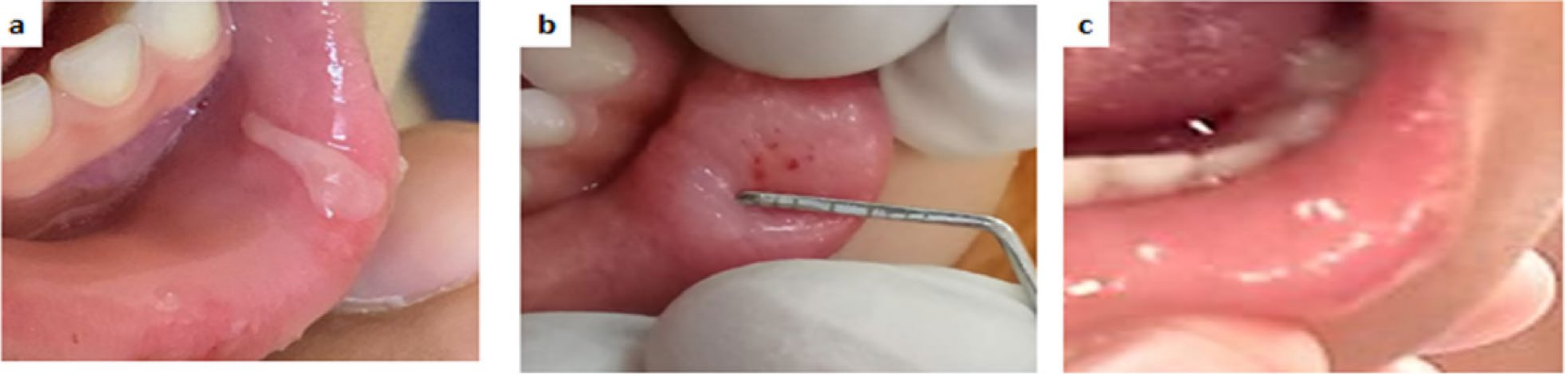
Clinical photos of a case of LC group illustrating the healing process at different time points; (**a**) pretreatment (baseline), (**b**) at day 5, (**c**) at day 7.

**Fig. 5:**
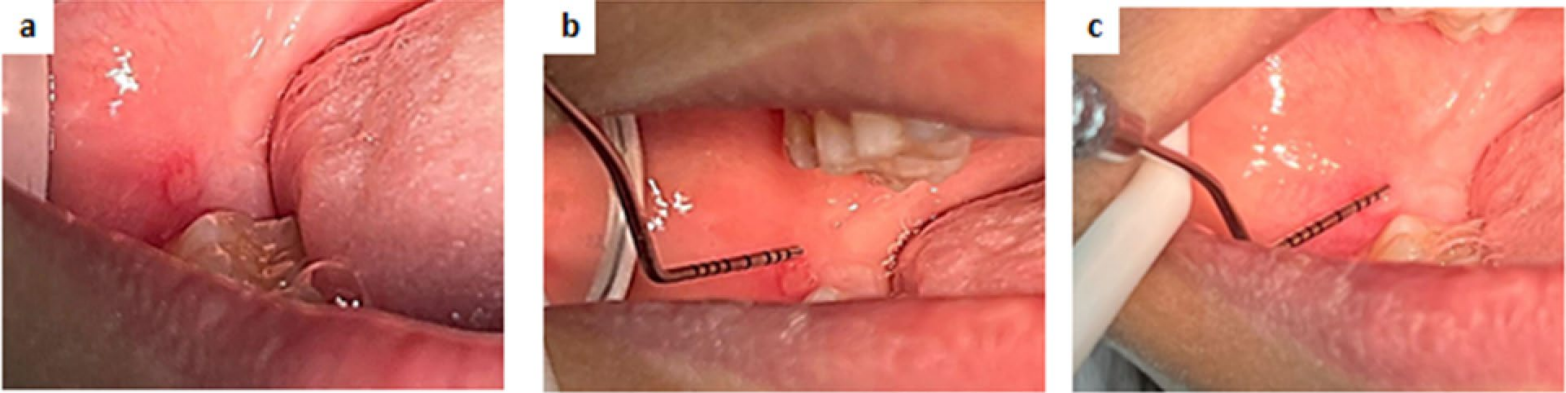
Clinical photos of a case of CHX group illustrating the healing process at different time points; (**a**) pretreatment (baseline), (**b**) at day 5, (**c**) at day 7.

Examples of clinical cases at different follow up periods are shown in figures (3, 4, 5).

### Statistical analysis

Statistical analysis was performed with SPSS 27^®^ (Statistical Package for Social Science, IBM, USA.), Graph Pad Prism^®^ (Graph Pad Technologies, USA) and Microsoft Excel 2016(Microsoft Co-operation, USA). All data were explored for normality by using Shapiro Wilk and Kolmogorov. Normality test which revealed that all data originated from non-parametric distribution (Except age), accordingly comparison between different groups was Kruskal Wallis test, while comparison between different time points was performed by using Friedman test, In age, comparison between groups was performed by using One Way ANOVA test. Regarding qualitative data, all comparisons were made by using Fisher’s Exact test and Chi square test. The correlation was assessed by using Spearman’s Correlation coefficient. The significant level was set to be at *P* ≤ 0.05.

## Results

### Baseline data (Table 1)

At baseline, the mean age of participants was comparable across the three groups, with Group I having a mean age of 11.5 ± 0.8 years, Group II at 11.8 ± 0.9 years, and Group III at 11.1 ± 0.7 years. The differences in age were not statistically significant (*P* = 0.16). Regarding sex distribution, Group I included 4 males (40%) and 6 females (60%), Group II had 6 males (60%) and 4 females (40%), while Group III consisted of 5 males (50%) and 5 females (50%). The distribution of sex across the groups also showed no significant difference (*P* = 0.67).

**Table 1:** Baseline data in all groups.

	Baseline data	Group I	Group II	Group III	*P* value
Age		11.5 ± 0.8	11.8 ± 0.9	11.1 ± 0.7	0.16
Sex	Male	4(40%)	6(60%)	5(50%)	0.67
	Female	6(60%)	4(40%)	5(50%)	

### Ulcer size

#### Intergroup comparison (comparison between groups)

**Fig. 6:**
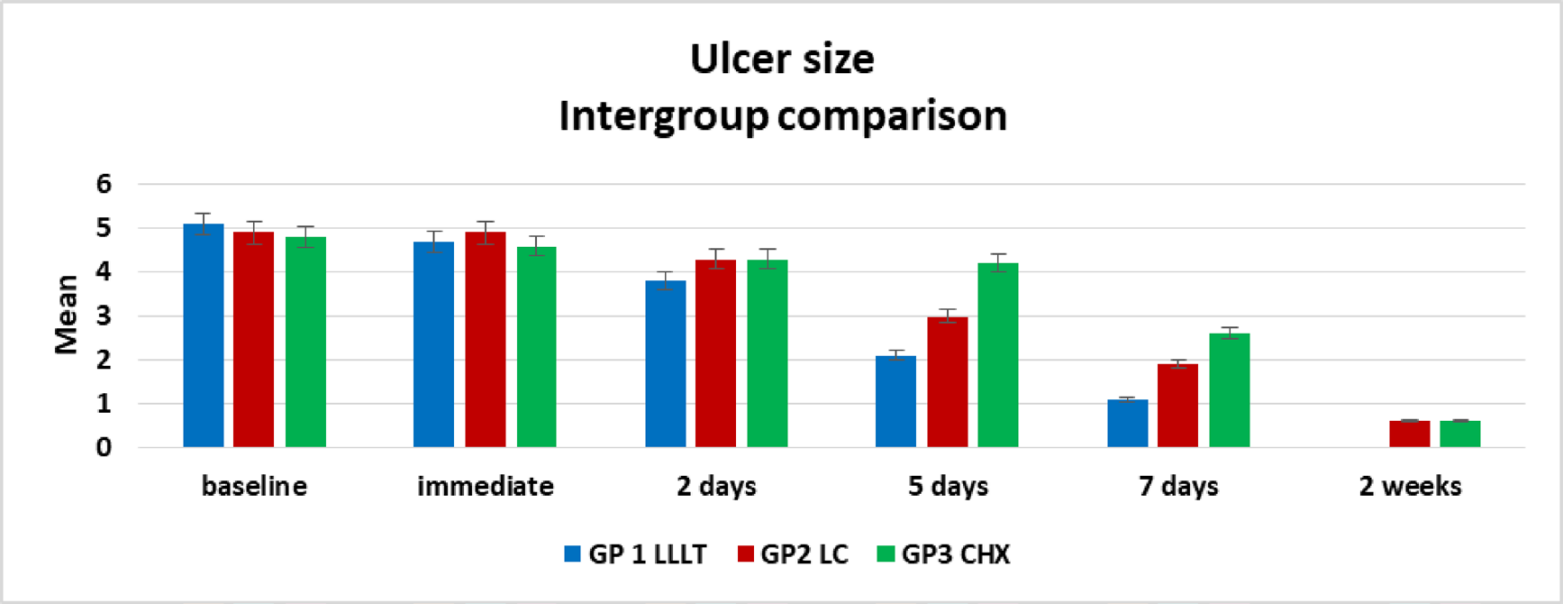
Bar chart showing intergroup comparison regarding ulcer size.

Descriptive data on ulcer size across all groups are presented in Table 2; Fig. 6. Analysis using the Kruskal–Wallis test revealed no statistically significant differences in ulcer size among Groups I, II and III at baseline, immediately after treatment, and after two days (*p* = 0.64, 0.74, and 0.22, respectively).

At the 5-day evaluation, ulcer size continued to decrease across all groups, with a statistically significant difference observed (*p* = 0.0001). Group I exhibited the greatest reduction, followed by Group II and then Group III. By day 7, healing had progressed in all groups, and a statistically significant difference remained (*p* = 0.0072). At this point, Group I demonstrated the smallest ulcer size, Group III the largest, while Group II showed an intermediate response that did not differ significantly from either Group I or Group III.

After two weeks, healing was nearly complete in all groups, although the difference remained statistically significant (*p* = 0.0080). Group I achieved complete healing in all cases, whereas Groups II and III exhibited minor residual ulcers in a few participants. The latter two groups were statistically comparable to each other but remained significantly different from Group I.

**Table 2:** Descriptive results of ulcer size in all groups, comparison between groups using Kruskal Wallis test.

		Minimum	Maximum	Median	Mean	Standard deviation	*P* value	Effect size
Baseline	Group I	4	6	5	5.10 ^**a**^	0.74	0.64	0.02
	Group II	4	6	5	4.90 ^**a**^	0.74		
	Group III	4	6	5	4.80 ^**a**^	0.79		
Immediate	Group I	3	6	5	4.70 ^**a**^	0.95	0.68	0.02
	Group II	4	6	5	4.90 ^**a**^	0.74		
	Group III	4	5	5	4.60	0.52		
2 days	Group I	3	5	4	3.80 ^**a**^	0.79	0.22	0.11
	Group II	3	5	4	4.30 ^**a**^	0.67		
	Group III	4	6	4	4.30	0.67		
5 days	Group I	1	3	2	2.10 ^**a**^	0.74	0.0001*	0.64
	Group II	2	4	3	3.00 ^**a**^	0.67		
	Group III	3	5	4	4.20 ^**b**^	0.63		
7 days	Group I	0	2	1	1.10 ^**a**^	0.74	0.0072*	0.35
	Group II	1	3	2	1.90 ^**ab**^	0.74		
	Group III	1	4	3	2.60 ^**b**^	1.07		
2 weeks	Group I	0	0	0	0.00 ^**a**^	0.00	0.0080*	0.33
	Group II	0	1	1	0.60 ^**b**^	0.52		
	Group III	0	1	1	0.60 ^**b**^	0.52		

*Significant difference as P < 0.05.

Means with different superscript letters were significantly different as P < 0.05.

#### Intragroup comparison (Comparison between intervals)

**Fig. 7:**
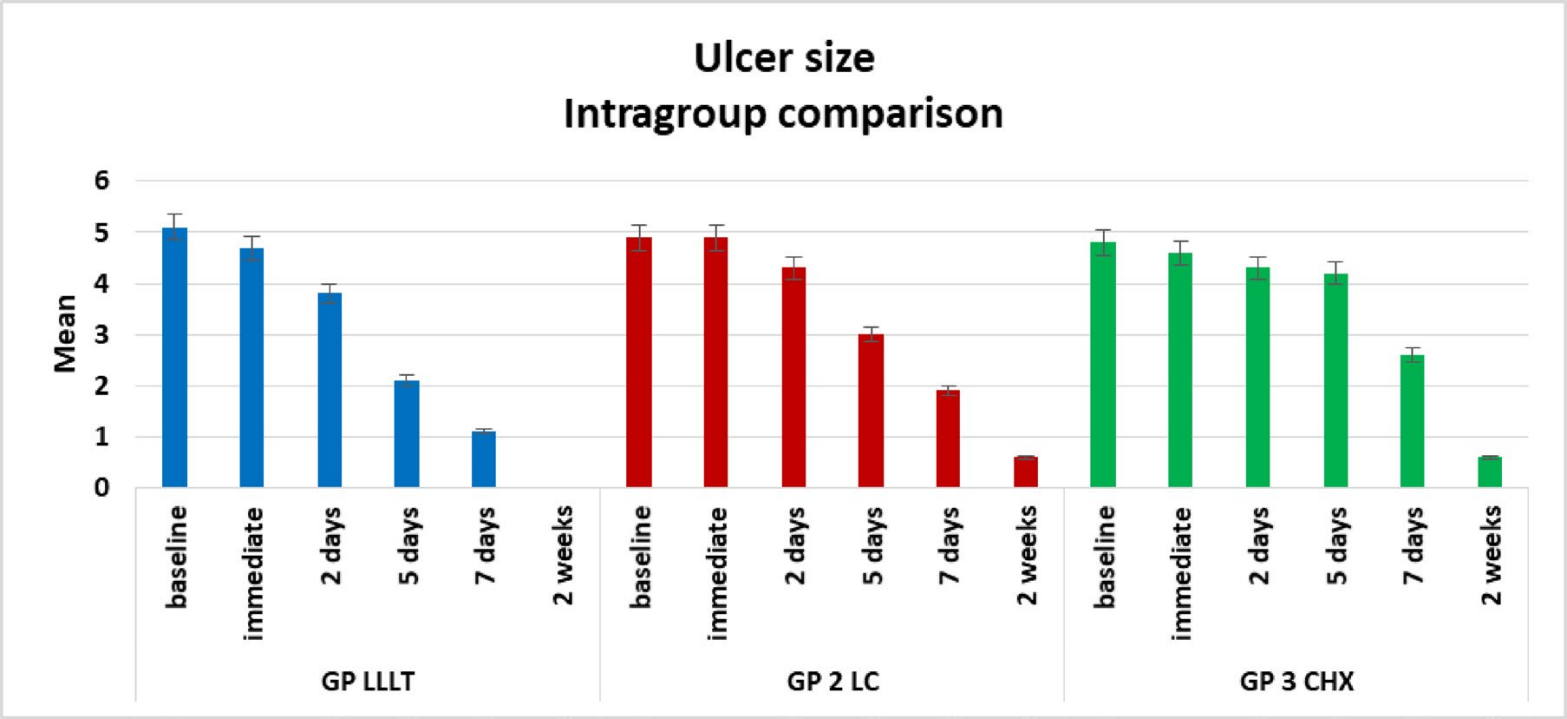
Bar chart showing intragroup comparison regarding ulcer size.

Descriptive data on ulcer size across all groups are presented in Table 3; Fig. 7. Comparison across time points using the Friedman test revealed the following:

- Group I: A statistically significant reduction in ulcer size was observed over time (*p* < 0.0001). A slight decrease occurred immediately after treatment, although this was not statistically significant. By day 2, the mean size continued to decrease, with significant reductions evident by days 5 and 7, culminating in complete healing at two weeks.
- Group II: A significant decline in ulcer size was also noted across time points (*p* < 0.0001). The mean size remained unchanged between baseline and immediately after treatment. Reduction commenced by day 2 but without statistical significance. More pronounced healing was observed by day 5, with significant reductions by day 7 and at two weeks, although complete healing was not achieved in all cases.
- Group III: A statistically significant improvement in ulcer size was observed over time (*p* < 0.0001), though healing progressed more slowly than in the other groups. The mean size decreased from baseline to day 7 without statistical significance, indicating minimal early response. By the second week, however, a significant reduction was detected.

**Table 3:** Descriptive results of ulcer size in all groups, comparison between different time points using Friedman test.

Ulcer size		Minimum	Maximum	Median	Mean	Standard Deviation	*P* value
Group I	baseline	4.00	6.00	5.00	5.10 ^**a**^	0.74	< 0.0001*
	immediate	3.00	6.00	5.00	4.70 ^**a**^	0.95	
	2 days	3.00	5.00	4.00	3.80 ^**ab**^	0.79	
	5 days	1.00	3.00	2.00	2.10 ^**b**^	0.74	
	7 days	0.00	2.00	1.00	1.10 ^**bc**^	0.74	
	2 weeks	0.00	0.00	0.00	0.00 ^**c**^	0.00	
Group II	baseline	4.00	6.00	5.00	4.90 ^**a**^	0.74	< 0.0001*
	immediate	4.00	6.00	5.00	4.90 ^**a**^	0.74	
	2 days	3.00	5.00	4.00	4.30 ^**a**^	0.67	
	5 days	2.00	4.00	3.00	3.00 ^**ab**^	0.67	
	7 days	1.00	3.00	2.00	1.90 ^**b**^	0.74	
	2 weeks	0.00	1.00	1.00	0.60 ^**b**^	0.52	
Group III	baseline	4	6	5	4.80 ^**a**^	0.79	< 0.0001*
	immediate	4.00	5.00	5.00	4.60 a	0.52	
	2 days	4.00	6.00	4.00	4.30 a	0.67	
	5 days	3.00	5.00	4.00	4.20 ^**a**^	0.63	
	7 days	1.00	4.00	3.00	2.60 **a**	1.07	
	2 weeks	0.00	1.00	1.00	0.60 ^**b**^	0.52	

*Significant difference as P ≤ 0.05.

Means with different superscript letters were significantly different as P ≤ 0.05.

### Pain assessment

#### Intergroup comparison

**Fig. 8:**
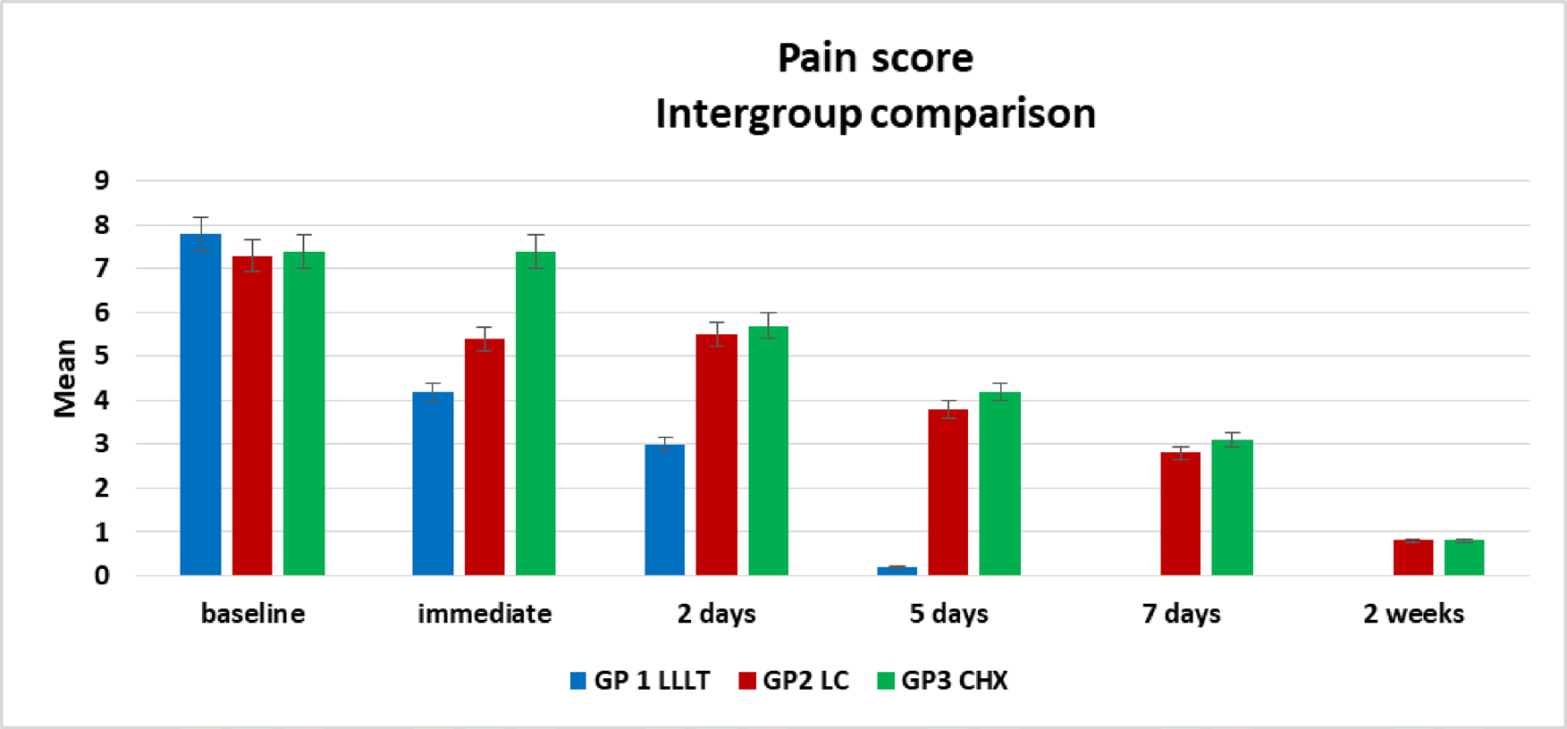
Bar chart showing intergroup comparison regarding pain score.

Descriptive data on pain scores across all groups are presented in Table 4; Fig. 8. Comparison between groups using the Kruskal–Wallis test revealed the following:

- Baseline: No statistically significant difference in pain scores was observed among the three groups (*p* = 0.2540).

Immediately after treatment and at day 2: A significant difference emerged between the groups (*p* = 0.0001). Group III reported the highest pain scores, significantly greater than those of Groups I and II, between which no significant difference was found at this time point. Similarly, after two days, Group I demonstrated significantly lower pain levels than Groups II and III, while the latter two remained statistically comparable.

- Days 5 and 7: Statistically significant differences persisted between groups (*p* = 0.0001). Group I reported minimal pain on day 5 and no pain by day 7. Groups II and III both showed reductions in pain but still reported significantly higher scores than Group I.
- Two-week evaluation: Differences in pain scores between groups remained statistically significant (*p* = 0.01), although pain had largely subsided in all groups. Group I reported no pain, whereas Groups II and III exhibited slight residual pain.

**Table 4:** Descriptive results of pain score in all groups, comparison between groups using Kruskal Wallis test.

Pain		Minimum	Maximum	Median	Mean	Standard Deviation	*P* value	Effect size
baseline	Group I	7.00	9.00	8.00	7.80 ^**a**^	0.79	**0.2540**	**0.12**
	Group II	7.00	8.00	7.00	7.30 ^**a**^	0.48		
	Group III	7.00	8.00	7.00	7.40 ^**a**^	0.52		
immediate	Group I	3.00	5.00	4.00	4.20 ^**a**^	0.63	0.0001*	**0.74**
	Group II	4.00	7.00	5.50	5.40 ^**a**^	1.17		
	Group III	7.00	8.00	7.00	7.40 ^**b**^	0.52		
2 days	Group I	2.00	4.00	3.00	3.00 ^**a**^	0.47	0.0001*	**0.67**
	Group II	4.00	7.00	5.50	5.50 ^**b**^	1.08		
	Group III	4.00	7.00	6.00	5.70 ^**b**^	1.06		
5 days	Group I	0.00	1.00	0.00	0.20 ^**a**^	0.42	0.0001*	**0.88**
	Group II	2.00	5.00	4.00	3.80 ^**b**^	0.92		
	Group III	3.00	5.00	4.00	4.20 ^**b**^	0.63		
7 days	Group I	0.00	0.00	0.00	0.00 ^**a**^	0.00	0.0001*	**0.84**
	Group II	2.00	4.00	3.00	2.80 ^**b**^	0.79		
	Group III	2.00	4.00	3.00	3.10 ^**b**^	0.74		
2 weeks	Group I	0.00	0.00	0.00	0.00 ^**a**^	0.00	0.01*	**0.27**
	Group II	0.00	2.00	1.00	0.80 ^**b**^	0.79		
	Group III	0.00	2.00	1.00	0.80 ^**b**^	0.79		

*Significant difference as P ≤ 0.05.

Means with different superscript letters were significantly different as P ≤ 0.05.

#### Intragroup comparison

**Fig. 9:**
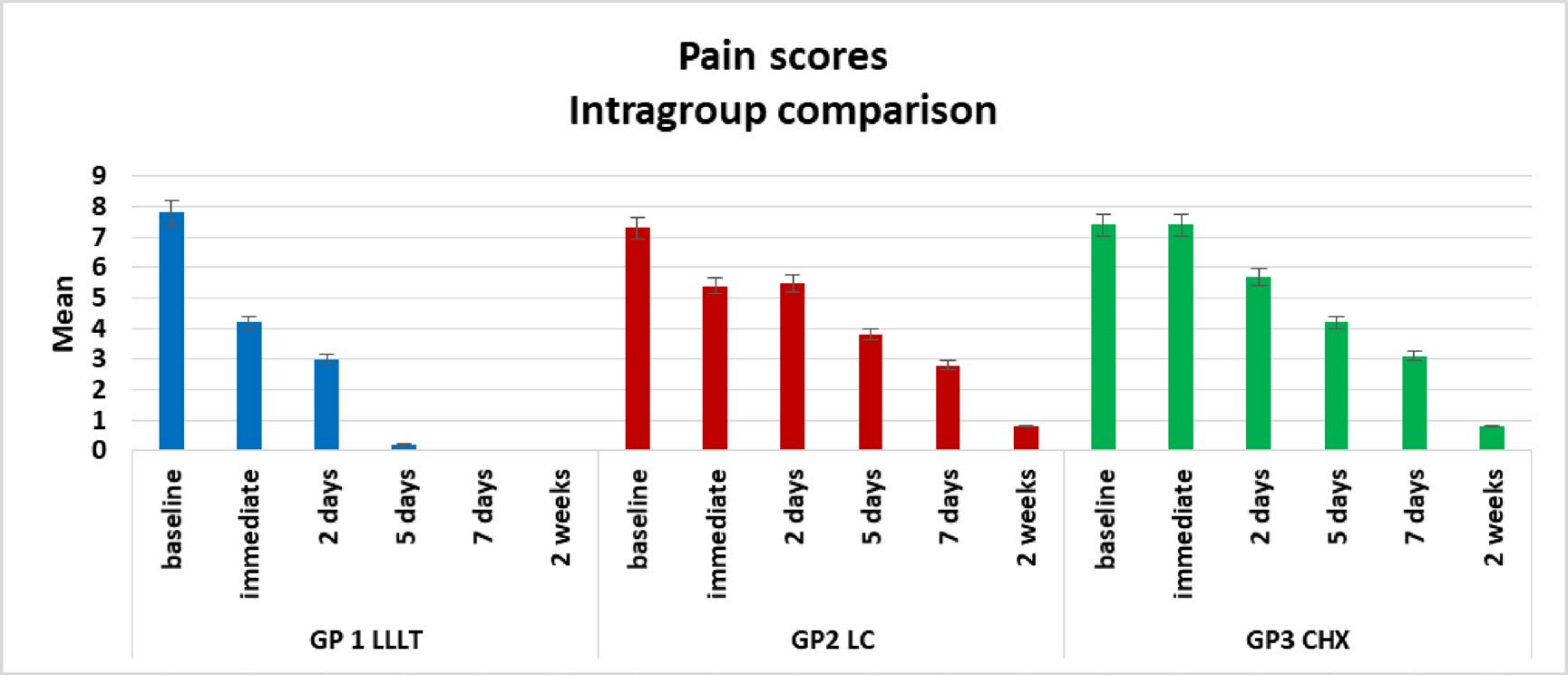
Bar chart showing intragroup comparison regarding pain score.

Descriptive data on pain scores across all groups are presented in Table 5; Fig. 9. Comparison across time points using the Friedman test revealed the following:

- **Group I: **Pain scores decreased significantly over time, reflecting rapid and consistent improvement following treatment. A reduction was noted immediately after treatment, although this change was not statistically significant. By day 2, the mean score had decreased further, with statistically significant reductions evident from day 5 onwards. Complete resolution of pain was achieved by day 7 and maintained throughout the two-week follow-up. Superscript letters confirm that scores from day 5 onwards were significantly different from baseline, indicating effective pain control in this group.
- **Group II: **Pain scores also demonstrated a statistically significant downward trend, although the improvement was more gradual than in Group I. The mean score decreased immediately after treatment and remained stable on day 2, with both values not significantly different from baseline, suggesting a delayed onset of relief. A clearer reduction was observed by day 5, with further improvement at one and two weeks. Superscript lettering confirms statistically significant improvements at later time points, although complete pain resolution was not achieved in all cases.
- **Group III: **Pain scores declined over time but at a slower rate compared with the other groups. The mean score remained unchanged from baseline to immediately after treatment, indicating no early response. A slight, non-significant reduction was seen by day 2, with more noticeable improvement by day 5. A statistically significant reduction occurred on day 7, and further decreases were observed by two weeks. These later improvements were statistically significant compared with earlier values, although the overall pattern indicated delayed and less complete relief relative to Groups I and II.

**Table 5:** Descriptive results of pain scores in all groups, comparison between different time points using friedman test.

		Minimum	Maximum	Median	Mean	Standard deviation
Group I	Baseline	7.00	9.00	8.00	7.80 ^**a**^	0.79
	Immediate	3.00	5.00	4.00	4.20 ^**a**^	0.63
	2 days	2.00	4.00	3.00	3.00 ^**ab**^	0.47
	5 days	0.00	1.00	0.00	0.20 ^**b**^	0.42
	7 days	0.00	0.00	0.00	0.00 ^**b**^	0.00
	2 weeks	0.00	0.00	0.00	0.00 ^**b**^	0.00
Group II	Baseline	7.00	8.00	7.00	7.30 ^**a**^	0.48
	Immediate	4.00	7.00	5.50	5.40 ^**ab**^	1.17
	2 days	4.00	7.00	5.50	5.50 ^**ab**^	1.08
	5 days	2.00	5.00	4.00	3.80 ^**bc**^	0.92
	7 days	2.00	4.00	3.00	2.80 ^**c**^	0.79
	2 weeks	0.00	2.00	1.00	0.80 ^**c**^	0.79
Group III	Baseline	7.00	8.00	7.00	7.40 ^**a**^	0.52
	Immediate	7.00	8.00	7.00	7.40 ^**a**^	0.52
	2 days	4.00	7.00	6.00	5.70 ^**a**^	1.06
	5 days	3.00	5.00	4.00	4.20 ^**ab**^	0.63
	7 days	2.00	4.00	3.00	3.10 ^**b**^	0.74
	2 weeks	0.00	2.00	1.00	0.80 ^**b**^	0.79

*Significant difference as P < 0.05.

Means with different superscript letters were significantly different as P < 0.05.

### Parental acceptance of the procedure

**Fig. 10:**
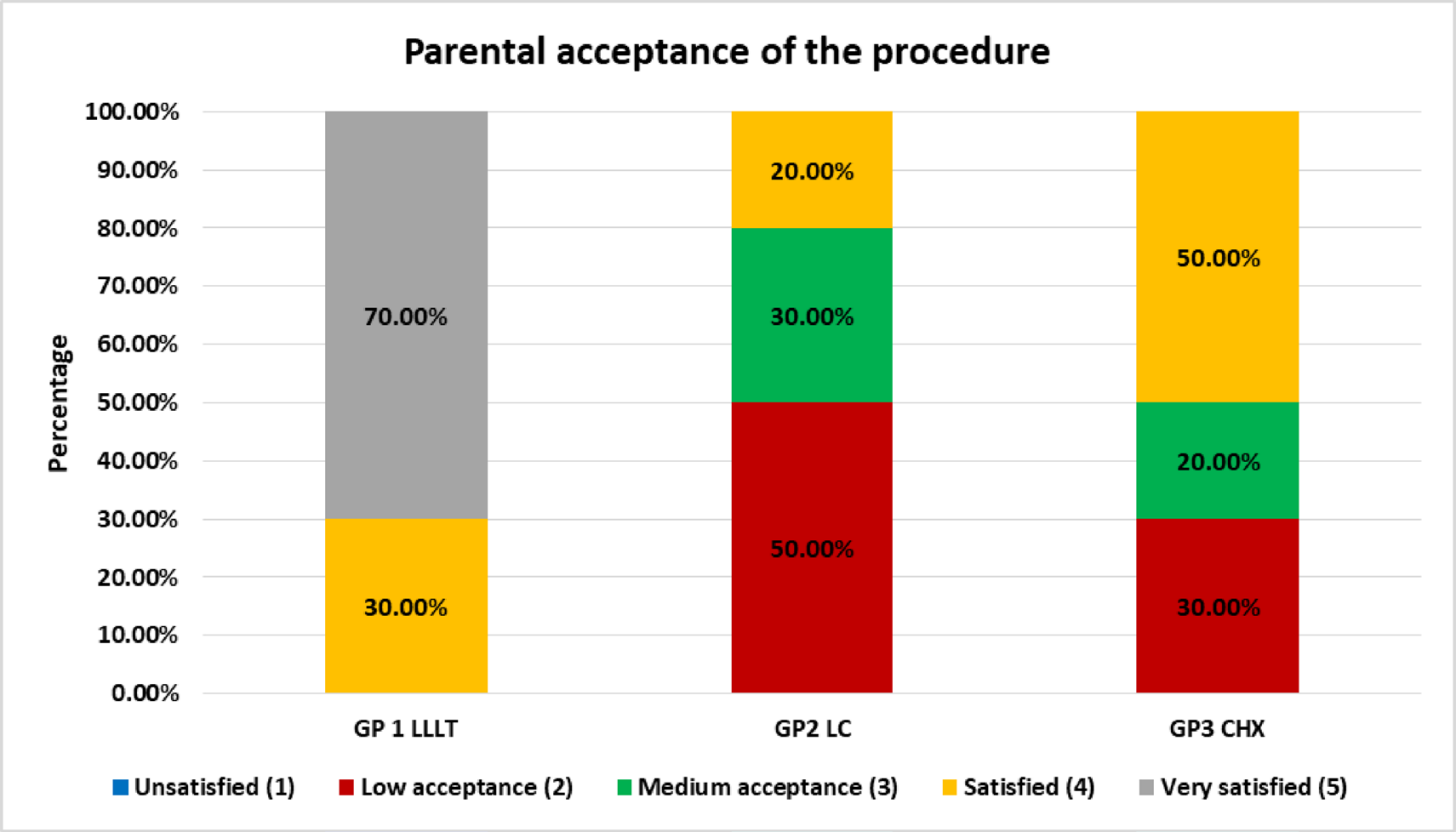
Stacked bar chart showing parental acceptance of the procedure.

Parental acceptance of the procedure across all groups is presented in Table 6; Fig. 10. Comparison between groups using Fisher’s Exact test revealed a statistically significant difference (*p* = 0.006).

- **Group I (LLLT): **Acceptance was overwhelmingly positive, with 70% of parents reporting being *very satisfied* and 30% *satisfied*. No parents expressed dissatisfaction or low acceptance, reflecting a high degree of approval and comfort with the laser-based approach.
- **Group II (LC): **A contrasting pattern was observed, with 50% of parents reporting low acceptance, 30% medium acceptance, and 20% satisfied. Notably, none rated the procedure as *very satisfied*, indicating limited parental confidence in this method.
- **Group III (CHX): **Responses were modest, with 30% of parents reporting low acceptance, 20% medium acceptance, and 50% satisfied. As in Group II, no parents rated the procedure as *very satisfied*. Although acceptance in this group was slightly higher than in the LC group, it remained considerably lower than in the LLLT group.

**Table 6:** Parental acceptance of the procedure regarding all groups.

Parental acceptance of the procedure	Group						P value
	Group I		Group II		Group III		
	N	%	N	%	N	%	P value
Unsatisfied (1)	0	0.0%	0	0.0%	0	0.0%	0.006*
Low acceptance (2)	0	0.0%	5	50.0%	3	30.0%	
Medium acceptance (3)	0	0.0%	3	30.0%	2	20.0%	
Satisfied (4)	3	30.0%	2	20.0%	5	50.0%	
Very satisfied (5)	7	70.0%	0	0.0%	0	0.0%	

*Significant difference as *P* < 0.05.

### Discomfort for the need of multiple appointments

**Fig. 11:**
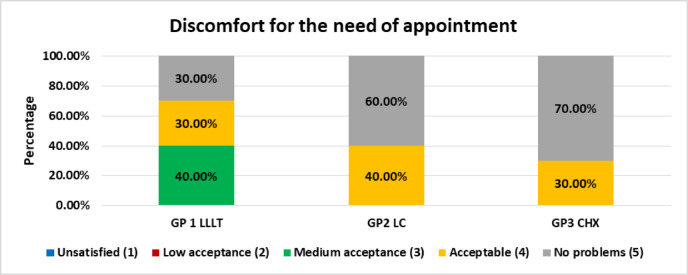
stacked chart showing Discomfort for the need of appointment.

Parental perceptions of discomfort associated with the need for multiple appointments across all groups are presented in Table 7; Fig. 11. Comparison between groups using Fisher’s Exact test revealed no statistically significant differences (*p* = 0.11), indicating that parental responses were broadly similar across treatment modalities.

- **Group I (LLLT): **Perceptions were more varied; 30% of parents reported no problems, 30% considered the requirement acceptable, and 40% rated it as medium acceptance. This distribution suggests some inconvenience in this group, though not of a severe nature.
- **Group II (LC): **A more favourable pattern was observed, with 60% of parents reporting no problems and 40% considering the schedule acceptable. No parents expressed low or medium acceptance, indicating a generally positive view of the appointment requirements.
- **Group III (CHX): **The highest proportion of parents (70%) reported no problems with the appointment schedule, while 30% rated it as acceptable. As in Group II, no parents expressed low or medium acceptance, suggesting broad parental comfort with the treatment schedule.

**Table 7:** Discomfort for the need of appointment regarding all groups.

Discomfort for the need of appointments	Group						P value
	Group I		Group II		Group III		
	N	%	N	%	N	%	
Unsatisfied (1)	0	0.0%	0	0.0%	0	0.0%	0.11
Low acceptance (2)	0	0.0%	0	0.0%	0	0.0%	
Medium acceptance (3)	4	40.0%	0	0.0%	0	0.0%	
Acceptable (4)	3	30.0%	4	40.0%	3	30.0%	
No problems (5)	3	30.0%	6	60.0%	7	70.0%	

### Correlation between ulcer size and pain

Table 8 presents the correlation between ulcer size and pain scores across the three study groups (GI, GII, and GIII). A statistically significant and strong positive correlation was observed in all groups, indicating that larger ulcer size was consistently associated with higher pain levels.

- **Group I (GI): **The correlation coefficient was *r* = 0.839 (*p* < 0.0001), reflecting a very strong and significant relationship between ulcer size and pain intensity. This indicates that increases in ulcer size were accompanied by notable increases in perceived pain.
- **Group II (GII): **An even stronger correlation was observed, with *r* = 0.858 (*p* < 0.0001), further reinforcing the association between greater ulcer dimensions and elevated pain levels, consistent with the findings in Group I.
- **Group III (GIII): **A strong positive correlation was likewise demonstrated, with *r* = 0.822 (*p* < 0.0001), confirming that increases in ulcer size were significantly associated with higher pain scores in this group as well.

**Table 8:** Correlation between ulcer size and pain.

		*r*	*P* value
Ulcer size and pain	GI	0.839	< 0.0001*
	GII	0.858	< 0.0001*
	GIII	0.822	< 0.0001*

*Significant correlation as *P* ≤ 0.05.

No harms or unintended events, such as ocular injury, mucosal burns, or medication-related toxicity, were reported in any group from baseline to the end of the follow-up period.

## Discussion

Oral ulcers resulting from dental trauma, whether acute or chronic, are common in children. The underlying aetiology is diverse and may involve thermal factors, such as the consumption of hot or cold foods, beverages, chemical agents, or irritation from orthodontic appliances. Physical factors are also recognised as significant oral ulcers contributors, including intentional or accidental self-biting and iatrogenic injury to the oral mucosa^23,24^.

Traumatic oral ulcers in paediatric patients are frequently associated with complications such as oral pain and impaired nutrition, both of which can negatively impact the child’s overall quality of life^6,25^.

The first step in management is the elimination of the causative or irritating factor. However, this step is not sufficient to ensure complete ulcer healing in a relatively shorter time. Keeping the ulcer without potential treatment may the affect the child oral quality of life in terms of pain sensation and discomfort during eating or drinking. Thereafter, topical therapy is commonly employed to alleviate symptoms and prevent secondary complications. Frequently used topical agents include CHX, benzocaine, LC, hyaluronic acid, topical antibiotics, and corticosteroids^3,26^.

Although chlorhexidine (CHX) is widely regarded as the gold standard for the management of oral ulcers, its use is not universally recommended. Chlorhexidine mouth wash is an anti-inflammatory medication which is highly potent against gram negative and gram positive oral pathogens. Because the mouthwash is retained in the oral cavity, it can completely cover the ulcer surface and give local disinfection effect. It has prolonged germicidal action at a physiological pH of 10. Benefits of CHX include decreasing plaque formation, reducing bacterial accumulation to the teeth, and altering the permeability of bacterial cell walls, which lead to cell lysis. Several authors have reported that CHX-based oral rinses may accelerate ulcer healing; however, they are often considered an unfavourable option in children due to side effects such as tooth and soft tissue discolouration, as well as their unpleasant taste^8,27^.

Advanced approaches, including the application of (LLLT), have been investigated in a limited number of recent studies on traumatic ulcer management in children. The mode of action of LLLT in the healing process of oral ulcer is related to enhancing the release of some analgesic agents like serotonin and endorphins. This mode occurs in line with reduction in prostaglandin synthesis giving anti-inflammatory effect with enhancing the vascular supply of the oral mucosa. Given its demonstrated ability to relieve pain, shorten healing time, and promote re-epithelialisation, LLLT is anticipated to gain wider clinical application^28,29^. A 980 nm laser system equipped with a flat-top beam profile handpiece was employed in the present study. This wavelength was selected for its advantageous deep tissue penetration properties, while the flat-top beam delivery system ensured homogeneous photon distribution across a 1 cm² target area. However potential side effects like ocular injury or mucosal burn could occur in the absence of protective eyeglasses or using incorrect wavelength^30,31^.

LC gel is a common, safe, and effective medication for local anesthesia. Nowadays, it is used topically for surface anesthesia of the skin or mucosa to alleviate ulcer pain till complete healing occurs. In our study, LC gel was applied in a 2% formulation, as this concentration has been established as both safe and effective in alleviating pain and reducing infection associated with paediatric oral ulcers. But adverse effects can follow LC improper use in the form of medication toxicity in cases of over dosage^32^. Similarly, a 2% concentration of CHX was utilised owing to its widespread use in both clinical practice and research. This aqueous formulation is generally considered biocompatible and possesses an acceptable toxicological profile, rendering it a reliable and effective treatment option for oral conditions^33,34^.

For ulcer size measurements, we adopted the ruler method using a calibrated periodontal probe, which enabled accurate estimation of ulcer size in millimeters. According to Majeske and Hanna et al., this method remains the most commonly used and valid tool for monitoring ulcer size as an indicator of healing^35^. Pain intensity was assessed using the (VAS), recognised as the gold standard for pain assessment. Its diagrammatic representation facilitates comprehension and allows children to more easily communicate their perceived level of pain^36^.

It is well established that children have a limited capacity to comprehend procedural details and the potential adverse effects associated with available treatment options. Consequently, they are generally unable to make informed choices between techniques or to provide rational acceptance of dental interventions. For this reason, parental acceptance was assessed using a Likert scale as a reliable proxy for evaluating a child’s satisfaction with the proposed treatment. To the best of our knowledge, however, few studies have examined parental satisfaction with the management of oral ulcers in children^37,38^.

Parental satisfaction represents a key objective in paediatric dental care. Treatment options are therefore often formulated following parental consultation, as parents are typically the primary decision-makers and seek to select what they perceive to be the most appropriate approach for their children if they are young to explain their feelings. At the same time, conveys their children’s acceptance or rejection towards particular treatment in cases of relatively older children^39,40^.

The objective of this study was to compare the healing of traumatic ulcers in children specifically in terms of pain reduction and ulcer size using (LLLT), LC gel, and (CHX) mouthwash. In parallel, parental satisfaction with these three treatment modalities was evaluated postoperatively.

Our results revealed that the mean age of participants was comparable across the three groups, with Group I having a mean age of 11.5 ± 0.8 years, Group II at 11.8 ± 0.9 years, and Group III at 11.1 ± 0.7 years. The differences in age were not statistically significant (*P* = 0.16), indicating good comparability among the groups in terms of age distribution. Regarding sex distribution, gender distribution was equal; 50% in each group (15 males and 15 females).The distribution of sex across the groups also showed no significant difference (*P* = 0.67), suggesting that the groups were well balanced demographically at baseline.

With respect to ulcer size, a well-recognised predictor of healing, our intergroup comparisons demonstrated a reduction in ulcer dimensions in Group I, followed by Group II and then Group III, with a statistically significant difference evident at the 5-day evaluation (*p* = 0.0001). By day 7, the healing process had progressed across all groups, although statistically significant differences persisted (*p* = 0.0072). At this stage, however, the rate of healing appeared comparable among the groups. To the best of our knowledge, few studies have directly compared these treatment modalities within a single trial.

Our findings are in agreement with a recent randomised controlled trial (RCT) which reported a greater reduction in ulcer size in the LLLT group compared with the LC group at day 3 []. Intragroup comparisons in our study further revealed a significant reduction in ulcer size in Group I from day 5 (*p* = 0.0001), with complete healing achieved by two weeks. Similarly, Group II demonstrated a significant reduction in ulcer diameter from day 5 onwards, although complete healing was not attained at two weeks. By contrast, Group III showed the largest ulcer sizes throughout the observation period, with only a significant reduction detected at the two-week evaluation. These negative findings of LC could be explained by its pharmacological components of pain reduction without any agent aid in wound healing.

These results align with another RCT, which demonstrated a significant reduction in ulcer size in the LLLT group compared with the control group (no intervention) from baseline through days 5 and 7 (*p* = 0.001)^41^. Likewise, a case report on paediatric oral ulcers treated with LLLT documented substantial reductions in ulcer dimensions at days 1, 3, and 7^11^, while another case report described complete healing by day 7^13^. In contrast, Domínguez et al. (2012) reported smaller ulcer diameters—and thus faster healing in a group treated with topical herbal medication (K-cit^®^) compared with LLLT, a discrepancy likely attributable to differences in participant age and the composition of the topical formulation^42^.

Regarding the healing pattern with CHX in our study, a gradual reduction in ulcer size was observed up to day 5, followed by a significant decrease at day 7 and complete resolution by two weeks. These findings are consistent with those of Sari and Mahendra (2023), who reported similar patterns in ulcer disappearance rates^43^. Another RCT likewise demonstrated slower reductions in ulcer size with CHX compared to an experimental intervention, although significant decreases were recorded at both day 3 and day 7 (*p* < 0.001)^44^. Differences between their findings and ours may be explained by the inclusion of older participants in their study, who may have been more compliant with CHX use than children.

Assessment of pain in the present study demonstrated a significant reduction in VAS scores across all groups. The LLLT group consistently exhibited the lowest scores at 2, 5, and 7 days (*p* = 0.0001), with complete pain resolution by two weeks (*p* = 0.01). In contrast, the LC and CHX groups showed residual pain at the end of the follow-up period. This difference in results could attributed to novelty of LLLT in relieving pain in shorter duration in comparison to CHX and LC.

This significant decline in VAS levels is consistent with the findings of Marya et al. (2021), who reported a reduction in VAS scores between days 1 and 3, with lower values in the LLLT group compared with the LC group^45^. Similarly, another study demonstrated significant pain reduction from day 3, with the LC group achieving lower scores than the CHX group^46^. Intra-group analyses in our study confirmed a significant reduction in VAS scores in the LLLT group at days 2 and 5, with complete pain relief by day 7. The analgesic effect of LLLT has been attributed to enhanced release of endogenous opioids and neurotransmitters, which suppress pain mediators and reduce the activity of C and A-delta fibres^47^.

These results for Group I are in agreement with Eroğlu et al. (2014), who reported reduced VAS scores from day 1 (*p* = 0.001) and significant reductions at days 3, 5, and 7 compared with untreated controls^41^. Comparable findings were also documented in earlier clinical studies, which demonstrated significant pain reduction in LLLT groups immediately after treatment and at day 3^11^, as well as pain elimination by day 4^13^ or gradual relief from day 4 until complete healing at day 8 in comparison with topical agents^42^.

In the LC group, a gradual reduction in VAS scores was observed, although the response was slower than in Group I. Pain reduction was apparent immediately after treatment, but values remained unchanged at day 2. Significant reductions were recorded at days 5, 7, and 14, although some participants still reported pain at the end of the follow-up period. This anti-inflammatory effect may be explained by the pharmacological properties of LC, including its ability to reduce TNF-α expression and inhibit the release of pro-inflammatory cytokines^46^. Similar outcomes were reported by Descroix et al. (2011)^9^, whereas Hopper et al. (2014) concluded that LC was less effective for pain control in children^32^, a discrepancy possibly explained by differences in inclusion criteria, as their study involved infected and painful oral ulcers.

The CHX group followed a similar overall pattern of pain reduction but at a slower rate. VAS scores remained stable immediately after treatment, with insignificant reductions at days 2 and 5. Significant improvements were not evident until days 7 and 14, and pain relief remained incomplete compared with Groups I and II. The analgesic action of CHX is thought to result from its antimicrobial properties and the direct contact of the mouthwash with the ulcer surface^48^. These findings are supported by a previous RCT, which reported significantly higher VAS values in the CHX group compared with an experimental treatment from baseline to day 7^49^. Similar pain relief patterns were observed in a recent case study^43^, although another clinical trial found no significant decline in pain with CHX^50^. Furthermore, significant reductions in VAS scores at days 3 and 5 were documented in a separate study, although levels remained higher than in the intervention group^44,51^. These variable outcomes may be attributed to differences in ulcer type, particularly studies focused on recurrent aphthous stomatitis rather than traumatic ulcers.

It is noteworthy to mention that it was challenging to assess the results of LC and CHX groups; which need child cooperation to apply the medication regularly. However every effort was done to motivate both parents and children to adhere to medications’ instructions, to gain their actual effect without influencing results.

With regard to parental satisfaction, LLLT achieved the highest level of acceptance, with no reported problems and an overall favourable attitude towards the minor inconvenience of multiple visits, when compared with the other groups. Relatively low parental acceptance to CHX and LC in comparison to LLLT could be attributed to their slower healing time and delayed reliving of annoying pain to their children caused by the traumatic ulcer.

These findings are consistent with a previous study that documented strong parental acceptance of laser therapy for the management of oral ulcers in children^18^.

Our results further demonstrated a statistically significant and strong positive correlation between ulcer size and pain intensity across all groups, indicating that larger ulcers are associated with higher pain scores. These two parameters therefore represent key predictors of the healing pattern of oral ulcers and may serve to guide clinical therapeutic decisions.

To the best of our knowledge, few studies have assessed parental satisfaction with different management strategies for paediatric oral ulcers, or examined the correlation between ulcer size and pain intensity, thereby underscoring the novelty of the present work.

In light of our findings, the null hypothesis was rejected, as significant differences were observed between LLLT, LC gel, and CHX mouthwash in relation to ulcer size reduction and pain alleviation. LLLT demonstrated the greatest therapeutic efficacy, whereas no significant difference was identified between LC gel and CHX mouthwash.

While the present findings provide valuable insights, certain limitations should be acknowledged. These include the relatively small sample size within each group and the inability to blind the clinical operator, the participating children, and their parents. Furthermore, the absence of histological evaluation limited the ability to elucidate the underlying mechanisms of healing associated with each intervention. Microbial load, therapeutic components of dentifrices, and secondary infection effects were not evaluated in this study however they can play a key role in oral wound healing. Future studies with larger sample sizes, studying all potential factors and the incorporation of histological analyses of healing traumatic ulcers in children are therefore recommended to strengthen the evidence base and clarify biological mechanisms.

## Conclusions

In conclusion, the findings of the present study demonstrate that LLLT provided the greatest efficacy in accelerating healing by reducing ulcer size and alleviating pain associated with traumatic oral ulcers, when compared with topical LC gel or CHX mouthwash. Despite its status as the conventional gold standard, CHX exhibited the lowest rates of healing and pain reduction. Furthermore, parental acceptance of LLLT was high, with minimal reported discomfort related to the required visits, in contrast to the other treatment groups.

## Data Availability

The datasets generated and/or analyzed during the present study can be obtained from the corresponding author upon reasonable request, with all precautions taken to safeguard participants’ privacy.
